# Right Renal Vein Fenestration: A Novel Anatomical Variant

**DOI:** 10.7759/cureus.95756

**Published:** 2025-10-30

**Authors:** Mugurel C Rusu, Nawwaf S Damen, Răzvan C Tudose, Adelina M Jianu

**Affiliations:** 1 Anatomy, Faculty of Dentistry, Carol Davila University of Medicine and Pharmacy, Bucharest, ROU; 2 Anatomy and Embryology, Faculty of Medicine, Victor Babeș University of Medicine and Pharmacy, Timisoara, ROU

**Keywords:** anatomic variation, fenestration, kidney, renal artery, renal vein

## Abstract

In the typical anatomy, two renal arteries, left (LRA) and right (RRA), enter the renal hilum, and two renal veins, left (LRV) and right (RRV), end in the inferior vena cava. Finding multiple bilateral renal arteries and veins is uncommon. The fenestration of the RRV has not been previously reported. We report it here in a variational context that includes multiple renal vessels and precaval RRAs. The anatomic variant reported here was identified during the evaluation of a 66-year-old male's CT angiogram. On each side, triple renal arteries were found, consisting of one main artery, superiorly, and two accessory arteries, middle and inferior. The main RRA was retrocaval, but the other two RRAs were precaval. All the renal arteries entered the renal hilum. A right inferior polar branch arose from the middle RRA. The RRV was doubled. The caval end of the inferior RRV was fenestrated. The anterior arm of this fenestration received the right gonadal vein. A circumaortic LRV was found. Its preaortic main arm was joined by the retroaortic one, which, in turn, drained the satellite veins of the middle LRA and inferior LRA. Various anatomical variants of the renal vessels may be found bilaterally and concomitantly. These should be carefully documented when surgical and endovascular interventions are planned. Knowledge of any possible morphological variations, whether frequent or extremely rare, is crucial.

## Introduction

The typical renal pedicle comprises two renal arteries, the left (LRA) and right (RRA), arising from the aorta and two renal veins, the left (LRV) and right (RRV), draining into the inferior vena cava (IVC). However, the number and course of these vessels vary frequently and can complicate retroperitoneal surgery and endovascular interventions [1]. For clarity, duplication denotes two distinct vessels with separate courses (e.g., two veins that do not rejoin), whereas fenestration refers to a single vessel that divides into two channels and later rejoins into one lumen. In addition, a precaval artery courses anterior to the IVC (contrasted with retrocaval, which runs posterior to it). These variants matter clinically because unrecognized additional vessels or unusual courses may increase the risks of hemorrhage, ischemia, or incomplete therapy during donor nephrectomy, renal transplantation, oncologic resection, varicocele or tumor embolization, and IVC or aortic reconstructions [1]. Fenestrations of the LRV are rare in the literature [2]. A multi-database search (PubMed, Embase, Scopus, Google Scholar) identified no indexed examples of RRV fenestration in humans. We report such a case here, occurring with multiple bilateral renal vessels and precaval RRAs [3].

## Case presentation

We retrospectively analyzed an archived angio-CT dataset from a 66-year-old man. The study complied with the World Medical Association’s Declaration of Helsinki and received approval from the relevant institutional authorities (affiliation 2; approval no. 16178/11 July 2023). Imaging was obtained using a 32-slice scanner (Siemens Multislice Perspective, Forchheim, Germany) using 0.6 mm collimation. Reconstructions were generated at 0.75-mm slice thickness with 50% overlap to produce multiplanar maximum-intensity projections. Post-processing was performed in Horos version 3.3.6 (Horos Project, Annapolis, MD, USA). All observations were verified on two-dimensional reformats and three-dimensional volume-rendered views by two separate reviewers.

**Figure 1:**
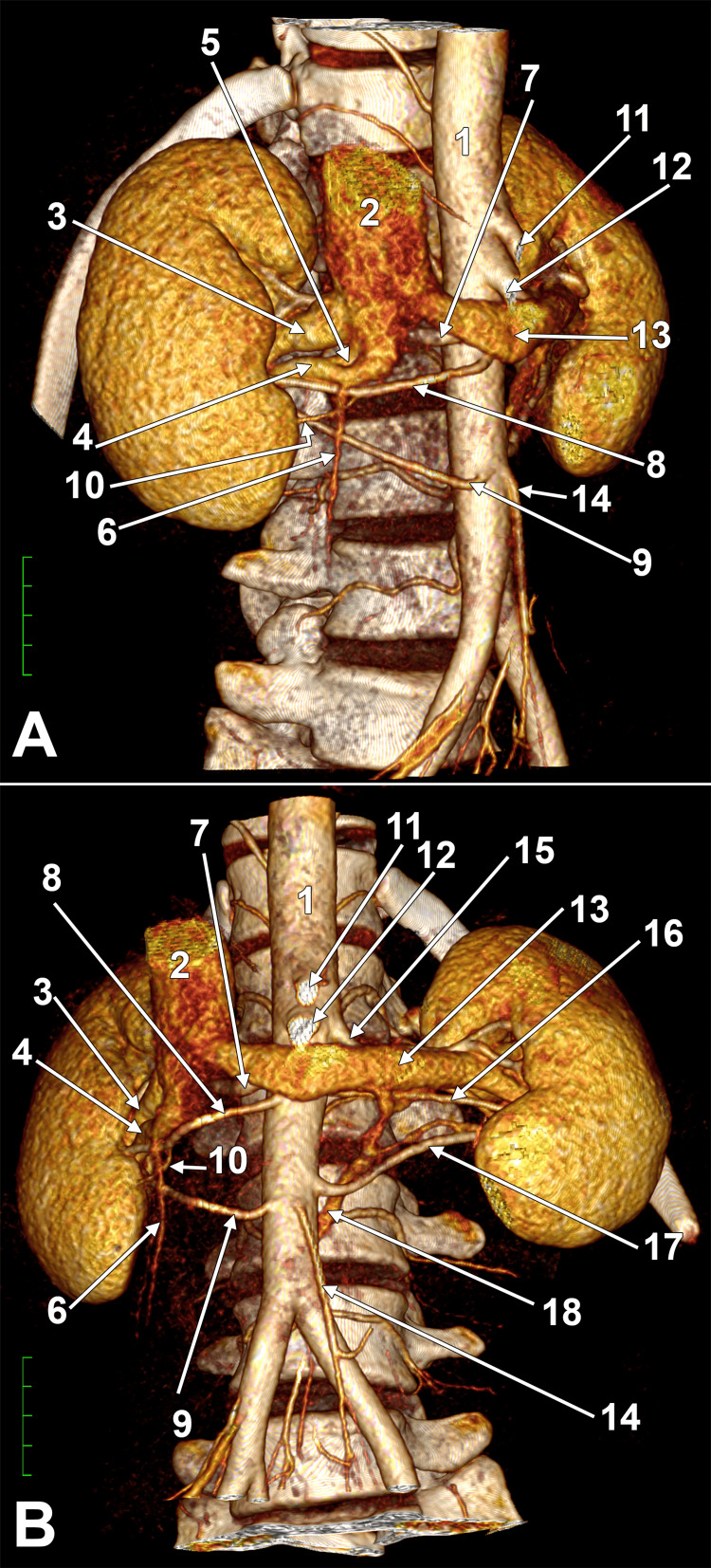
Double RRVs. Fenestrated inferior RRV. Bilateral triple renal arteries. Circumaortic LRV. Three-dimensional volume renderings. (A) Right anterior view. (B) Anterior view. 1. aorta; 2. IVC; 3. superior RRV; 4. inferior RRV; 5. fenestration of the inferior RRV; 6. right gonadal vein; 7. superior RRA; 8. precaval middle RRA; 9. precaval inferior RRA; 10. inferior polar branch; 11. celiac trunk; 12. superior mesenteric artery; 13. preaortic LRV; 14. inferior mesenteric artery; 15. superior LRA; 16. middle LRA; 17. inferior LRA; 18. retroaortic LRV. RRV: right renal vein; LRV: left renal vein; IVC: inferior vena cava; RRA: right renal artery; LRA: left renal artery.

On the right side, three RRAs were identified. The most cranial vessel, the dominant RRA (0.35 cm), arose opposite the upper third of L2 and coursed retrocavally (posterior to the IVC), passing posterior to the upper RRV. Two additional accessory RRAs followed precaval paths: the middle RRA (0.18 cm) originated from the aorta at the lower third of L2, issued an inferior polar branch, and proceeded to the right renal hilum; the lowest RRA (0.16 cm) arose from the aorta at mid-L3, passed posterior to the inferior polar branch, and entered the hilum (Figure 1). The inter-ostial distances between RRAs were 0.71 cm (upper-middle) and 3.27 cm (middle-lower).

**Figure 2:**
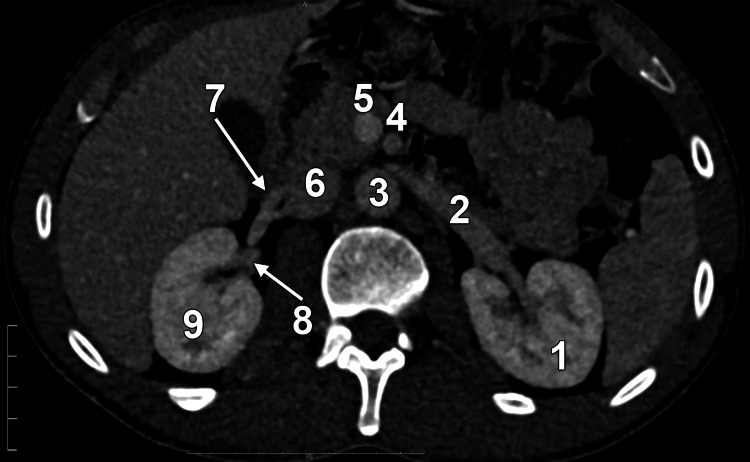
Axial slice through the kidneys, inferiorly viewed. 1. Left kidney; 2. LRV; 3. aorta; 4. superior mesenteric artery; 5. portal vein; 6. IVC; 7. fenestrated inferior RRV; 8. superior RRV; 9. right kidney. LRV: left renal vein; IVC: inferior vena cava; RRV: right renal vein.

Two RRVs entered the IVC at the level of the upper third of L2. The upper RRV measured 0.90 cm in diameter. At 1.19 cm proximal to its IVC confluence, the lower RRV (0.54 cm) bifurcated into an anterior arm (0.35 cm) and a posterior arm (0.46 cm), creating a fenestrated segment (Figure 2). The fenestration window measured 0.49 cm transversely and 0.28 cm sagittally. The right gonadal vein drained into the anterior arm of the fenestration and was crossed posteriorly by the middle RRA and its inferior polar branch (Figure 1).

On the left side, three LRAs were present: the superior (main) LRA (0.21 cm) originated at the L1-L2 intervertebral disc level; the middle LRA (0.14 cm) arose from the middle third of L2; and the inferior LRA (0.24 cm) arose from the upper third of L3. All LRAs entered the left renal hilum (Figure 1). Inter-ostial distances between LRAs were 1.04 cm (upper-middle) and 2.23 cm (middle-lower).

Two LRVs configured a circumaortic morphology: the preaortic LRV measured 1.13 cm, and the retroaortic LRV measured 0.35 cm. The retroaortic LRV followed a retroaortic course at the lower third of L3, received a satellite vein of the inferior LRA, and connected superiorly with the satellite vein of the middle LRA at 0.78 cm proximal to its confluence with the main, preaortic LRV (Figure 1).

## Discussion

Around the eighth week, the bilaterally symmetrical cardinal venous system converts into the right-sided IVC [4]. At this time, two renal veins, ventral and dorsal, are present on each side [4]. These will further coalesce into a single one [4]. If they do not fuse, the double veins will remain [4]. Supernumerary RRVs are more common because the right venous shift of the inferior vena cava system is unsupportive for the retention of additional LRVs [4].

Few authors found and reported fenestrated LRVs [5-7]. Bergman’s comprehensive Encyclopedia of Human Anatomic Variation [8] does not list the fenestrations of the renal veins. To the authors’ knowledge, the fenestration of the RRV is an anatomical novelty.

A meta-analysis on the renal veins established a 13.8% prevalence for the double RRVs and a 1.7% prevalence for the triple RRVs [9]. In cases with fenestrations of the RRV, the arms of the fenestration could mimic a double vein. Fenestrated RRVs could be accurately discriminated tridimensionally.

The right gonadal vein typically drains into the ICV. The drainage of the right gonadal vein into the RRV was found in 6.2% of cases. As fenestrated RRVs were not reported previously, we may assume that neither was a right gonadal vein draining into a fenestrated RRV.

Retroaortic LRVs are topographically variable [10]. A circumaortic LRV has preaortic and retroaortic components, such as in our case. The overall prevalence of this variant is 3.5% [9]. Embryologically, the circumaortic renal vein develops from the persistence of the intersupracardinal venous anastomosis, left supracardinal anastomosis, and dorsal left renal vein [11].

We found bilateral triple renal arteries here. On each side, the upper one was the main one, while the other two were accessory arteries. The two accessory RRAs had precaval courses. A recent meta-analysis of the renal arteries determined a 0.30% prevalence of bilateral triple renal arteries [12], similar to our case. A double precaval RRA, as reported here, was found to have a prevalence of 0.50% [13].

The left kidney is preferred for transplantation due to its longer pedicle. The right kidney may be an alternative when the left one is morphologically improper. Multiple renal vessels may complicate surgical access and require careful preoperative planning. In cases with bilateral multiple renal vessels, such as in this report, it is difficult to decide which kidney is better suited for transplantation. If nephrectomies are chosen in such cases with multiple renal arteries and veins, detailed preoperative imaging studies are indispensable for preoperative planning and a safe retroperitoneal approach.

## Conclusions

This case demonstrates the remarkable complexity of congenital renal vascular variants, with the unprecedented combination of bilateral triple renal arteries, precaval accessory RRAs, doubled RRVs with fenestration, and circumaortic LRV. The fenestration of the RRV represents a previously unreported anatomical variant that adds to our understanding of renal vascular embryological development and morphological diversity. The clinical implications of such complex anatomy are substantial, particularly for surgical and endovascular interventions involving the renal vasculature. Comprehensive preoperative imaging with detailed vascular mapping is essential for safe surgical planning, as these variants can significantly alter operative approaches and increase procedural complexity. Knowledge and recognition of the full spectrum of renal vascular anomalies, including extremely rare variants like RRV fenestration, remain crucial for optimal patient outcomes in urological, transplant, and vascular surgical procedures.
